# miRNA-mediated deregulation in leukemia

**DOI:** 10.3389/fgene.2012.00252

**Published:** 2012-11-15

**Authors:** Carmela Dell’Aversana, Lucia Altucci

**Affiliations:** ^1^Institute of Genetics and Biophysics, Consiglio Nazionale delle RicercheNaples, Italy; ^2^Department of General Pathology, Second University of NaplesNaples, Italy

**Keywords:** hematopoiesis, leukemia, microRNA

## Abstract

MicroRNAs (miRNAs) are small non-coding RNAs 18–25 nucleotides (nt) long able to fine-tune post-transcriptional gene expression. Extensive investigation into biogenesis, mechanism of action and functions of miRNAs has clearly revealed their prompt control in developmental timing, differentiation, proliferation, cell death, and metabolism. Deregulation of miRNA-mediated pathways may contribute to pathological conditions such as tumors, including hematological cancers, thus suggesting that miRNAs act both as tumor-suppressor genes (TSG) and oncogenes (OG). Here, we provide an overview of the current understanding of the aberration of miRNA biogenesis, activity, and post-transcriptional control in leukemogenesis.

## miRNA BIOGENESIS AND FUNCTION

MicroRNAs (miRNAs) are small, 18–25 nucleotide-long non-coding RNA molecules, known to be key regulatory elements in a wide range of biological functions. The first to be described, in the early 1990s, were lin-4 and let-7, regulators of developmental timing in *Caenorhabditis elegans *(Lee et al., 1993;Wightman et al., 1993), initially called small temporal RNAs (stRNAs;Pasquinelli et al., 2000) and later renamed miRNAs (Lagos-Quintana et al., 2001). In the last two decades a considerable, though only partial, understanding of the fine regulation operated by and modulated on miRNAs has been achieved. The biogenesis of mature miRNAs begins from transcription of primary transcripts called pri-miRNAs by RNA polymerase II. miRNA transcription can be related to independent miRNA-coding gene units, non-coding RNA transcripts, and protein-coding genes. About 50% of miRNAs are expressed as polycistronic miRNAs in clusters under the control of a unique promoter. Several miRNA genes show comparable characteristics to protein-coding genes, such as the relative incidence of CpG islands, TATA box, TFIIB recognition, initiator elements, and histone modifications (Ozsolak et al., 2008;Corcoran et al., 2009). Primary miRNA transcripts are characterized by a hairpin RNA structure with 7-methyl-guanylate (m7G) cap at 5′-end, a poly(A) tail, and may include introns (Czech and Hannon, 2011). In the nucleus, pri-miRNAs are recognized by a microprocessor complex containing the ribonuclease Drosha, its cofactor DiGeorge critical region 8 (DGCR8) and several other factors such as EWSR1, FUS, numerous heterogeneous nuclear ribonucleoproteins (hnRNPs), p68 (DDX5) and p72 (DDX17) DEAD-box helicases (Gregory et al., 2004). The Drosha complex recognizes the terminal hairpin loop (≥ nt) and cleaves 22 nt, or 2 helix turns, from the terminal loop/stem junction by cropping (Zeng and Cullen, 2004). The resulting shortened hairpin structure (pre-miRNA) comprises about 70 nt with a 2 nt 3′ overhang and a phosphate group at the 5′-end (Han et al., 2006;Seitz and Zamore, 2006). However, recent findings have identified some miRNAs, called mirtrons, located within intron regions of protein-coding genes, which bypass the Drosha processing step using intronic splicing to produce pre-miRNAs (Okamura et al., 2007;Ruby et al., 2007). pre-miRNAs are then translocated into cytoplasm by exportin 5 (Exp-5) in cooperation with Ran-GTP61 (RAS-related nuclear protein with bound GTP). Exp-5 specifically interacts with double-stranded RNAs of least 16 bp facilitated by 3′ overhang and, after GTP hydrolysis, releases pre-miRNAs (Bohnsack et al., 2004;Zeng and Cullen, 2004). In cytoplasm, processed miRNA precursors are cleaved by another type-2 RNase III enzyme, Dicer, to produce ~22 nt duplex miRNAs with 3′ overhang (Kim et al., 2009). From the resulting miRNA duplex, the mature miRNA guide strand is loaded onto Ago (Argonaute)-2 protein with Dicer and TRBP (HIV transactivating response RNA-binding protein), forming the so-called miRNA-induced silencing complex (miRISC), while the passenger strand is usually degraded (Bartel, 2009) or, in some cases, can become functional miRNA (Kuchenbauer et al., 2011). miRNA-modulated gene regulation results in a complex post-transcriptional mechanism mediated by the complementarity between the “seed” sequence (positions 2–8 from the 5′-end of the miRNA) and the “seed-match” sequence (generally in the 3′UTR of the target mRNA). The inhibitory function of miRNAs can occur either via translational repression or mRNA degradation, depending on the lesser or greater degree of miRNA/mRNA complementarity respectively (Filipowicz et al., 2008). Moreover, miRNAs may target DNA or hnRNPs, or increase expression of a target mRNA (Garzon et al., 2010). To date, approximately 1800 human miRNAs have been identified (http://www.mirbase.org/) and over one third of human genes are putative miRNA targets (Croce, 2009).

## miRNAs AS ACTORS IN NORMAL HEMATOPOIESIS

**FIGURE 1:**
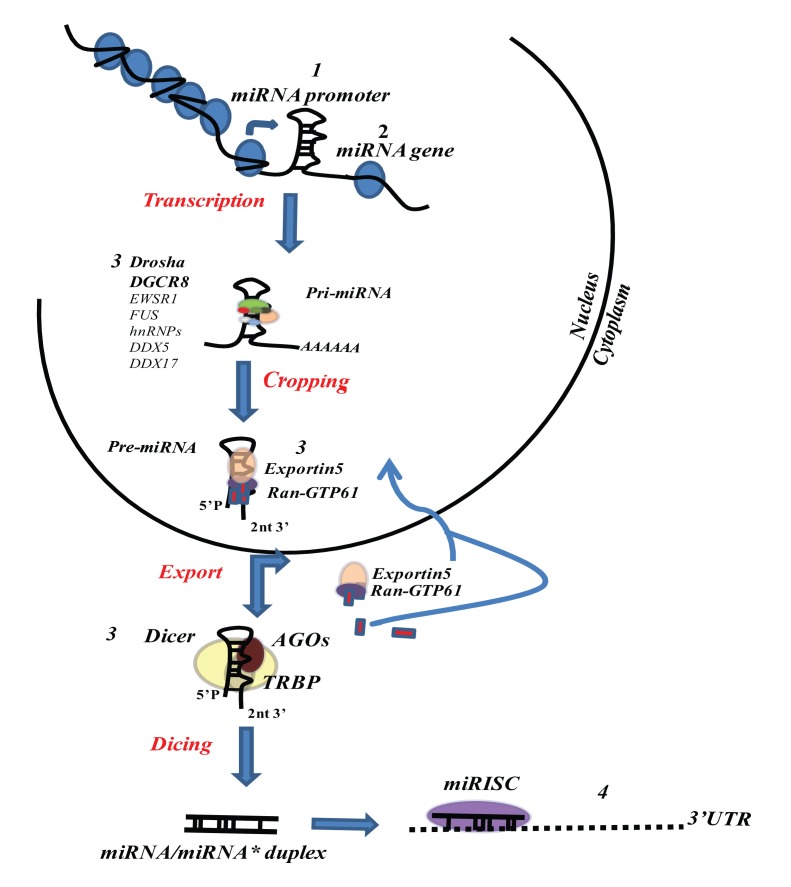
**Mechanism of miRNA expression and/or function perturbation**. **(1)** Epigenetic changes (DNA methylation and histones modifications); Transcription factor or regulatory gene alteration. **(2)** Genomic changes (deletions, amplifications, translocation, SNPs, mutations). **(3)** Functional or expressional biogenesis pathway alterations (Drosha down-regulation; XPO5 mutation; DISC assembly defects). **(4)** Alteration of target mRNAs 3′UTR.

Strongly conserved among distantly related organisms, miRNAs are involved in a variety of biological processes including cell cycle regulation, apoptosis, differentiation, development, metabolism, and aging (Lujambio and Lowe, 2012). Hence, deregulation of miRNA networks seems to contribute to malignant transformation. The causes of altered miRNA expression and/or function are disparate and include deletion, amplification, mutation, transcriptional deregulation, and epigenetic changes, which may involve miRNAs directly or their regulatory factors (Ryan et al., 2010; **Figure 1**).

Recent studies have emphasized the fine-tuning of gene expression by several miRNAs in the hematopoietic system and the clear relationship between imbalance of miRNA profiles and leukemic phenotype (O’Connell et al., 2010a). In the hematopoietic system, a wide set of highly specialized cells are produced from a common stem cell population by a hierarchical differentiation process. miRNAs have been shown to be key supporting actors in molecular control networks of hematopoiesis, including lineage decisions, stem cell progenitor transitions, niche control and other cell functions (O’Connell et al., 2010b;O’Connell and Baltimore, 2012). To highlight their importance in hematopoiesis, *in vivo* studies in conditional knockout mice were performed, given that Dicer knockouts are embryonic lethal (Bernstein et al., 2003). Likewise, DGCR8-deficient embryonic stem (ES) cells are blocked in G1 phase and exhibit defective differentiation (Wang et al., 2007, 2008). Furthermore, Ago2 inactivation causes significant hematopoietic defects (O’Carroll et al., 2007).

Several miRNAs play a critical role in stem/progenitor, lymphoid, myeloid, erythroid, and megakaryocytic biology, and in the immune function of these cell lineages (O’Connell et al., 2010b). Individual miRNAs are essential in the maintenance, differentiation, and control of lineage determination of ES cells. miRs-290–295, miR-296, miR-21, and miR-22 are increased following induction of differentiation (Gangaraju and Lin, 2009;Wang et al., 2009). The key ES cell transcription factors, Oct4, Sox2, Nanog, and Tcf3, are associated with miRNA promoters preferentially expressed in ES cells, such as miR-290 cluster (Marson et al., 2008). miR-134, miR-296, and miR-470 up-regulated on retinoic acid (RA)-induced differentiation, target Nanog, Oct4, and Sox2, leading to transcriptional and morphological changes characteristic of differentiating mouse ES cells (Tay et al., 2008). miR-196b is most abundantly expressed in short-term hematopoietic stem cells (HSC), but is downmodulated in progenitors (Popovic et al., 2009). Moreover, miR-150 is involved in cell fate decisions by tuning MYB levels in mixed erythroid/megakaryocytic progenitors (EMP); high miR-150 expression triggers megakaryoid (Mk) differentiation, while low miR-150 expression favors erythroid differentiation (Lu et al., 2008). miR-10a, miR-10b, miR-17, miR-20, miR-106, and miR-126 are down-regulated during Mk differentiation. Interestingly, the regulatory circuitry of miR-223 is implicated in myelopoiesis: nuclear factor I-A (NFI-A) maintains miR-223 at low levels, whereas after RA-induced differentiation C/EBP alpha up-regulates miR-223 expression, both acting via CCAAT-box binding on miR-223 promoter (Fazi et al., 2005). Increase of miR-27 expression is required to downmodulate AML1 expression during granulocytic differentiation (Feng et al., 2009). In the Mk lineage, miR-28 has been shown to inhibit differentiation by targeting thrombopoietin receptor (TpoR;Girardot et al., 2010). AML1 itself controls monocytopoiesis in a mutual negative feedback loop with miRNA 17-5p-20a-106a: AML1 binds the miRNA 17-5p-92 and 106a-92 cluster promoters and transcriptionally inhibits their expression; miRNA 17-5p-20a-106a suppresses AML1 protein expression, leading to blast proliferation and inhibition of monocyte differentiation and maturation (Fontana et al., 2007). miR-125b supports myelopoiesis, but not G-CSF-induced granulocytic differentiation, by regulating c-Jun and JunD pathways (Surdziel et al., 2011). For erythroid differentiation of CD34+ cells, miR-221 and miR-222 are downmodulated and unblock kit protein production at mRNA level (Felli et al., 2005). Expression of miR-451 is significantly up-regulated during erythroid maturation (Zhan et al., 2007). In normal human CD34+ cells, miR-15a and miR-16-1 can repress c-Myb expression with a negative autoregulatory feedback loop between c-Myb and the miR-15a/miR-16-1 cluster (Zhao et al., 2009). miR-144/451 cluster is controlled by transcription factor GATA-1, a master regulator of erythroid cell development (Orkin and Zon, 2008). miR-24 has been shown to down-regulate erythropoiesis by targeting hALK4, reducing activin-mediated Smad2 phosphorylation and attenuating transcriptional responses of activin (Wang et al., 2008). Convincing evidence has demonstrated that severe impairment of miRNA regulatory mechanisms affect immune development/response leading to immune disorders (Ha, 2011). miRNA functions are essential to maintain immune homeostasis. For example, overexpression of miR-34a leads to disruptions in B-cell development by repressing Foxp1 (Rao et al., 2010). Knockout of miR-155 affects T-cell differentiation, germinal center B-cell responses, and responses to bacterial and viral infection (O’Connell et al., 2007;Rodriguez et al., 2007;Thai et al., 2007). miR-146a plays a key role in innate immunity, inflammatory response, and viral infection. Both TLR activation and inflammatory stimulation can lead to up-regulation of miR-146a, which in turn negatively regulates innate immunity by repressing its targets, such as IRAK1, IRAK2, TRAF6, and TBP (Taganov et al., 2006;Hou et al., 2009;Sinha et al., 2010). By inhibiting miR-155 expression progesterone can down-regulate both MyD88 and TRIF-dependent signaling pathways as well as IL-6 and IFN-β production (Sun et al., 2012b). Recent studies reported that miR-23b induces tolerogenic dendritic cell (DC) activity and Treg responses *in vitro* through inhibition of the Notch1 and NF-κB signaling pathways (Zheng et al., 2012). miR-155-deficient myeloid DCs have an impaired ability to trigger T-cell activation after antigen presentation (Rodriguez et al., 2007). In the lymphocyte lineage, the mir-17-92 cluster is highly expressed in B- and T-precursor cells and its expression diminishes after maturation. The loss of miR-17/miR-19b causes protein-level changes of TGF-βRII, CREB1 and Pten and affects thymus selection of the nTreg population (Jiang et al., 2011). Finally, miR-125b is able to activate immune responses of macrophages by reducing IRF4 levels (Chaudhuri et al., 2011). Taken together, these findings underscore the fundamental role of miRNAs in the synergic regulation of pathways, such as cell fate decisions, development and differentiation of hematopoietic and immune cells.

## CRUCIAL ROLE OF miRNAs IN LEUKEMOGENESIS

Leukemogenesis is a complex process that involves multiple genetic and epigenetic events. It underlies a group of clonal malignancies of blood and bone marrow characterized by the presence of chromosomal abnormalities, such as deletions, translocations or inversions, or genetic mutations affecting the control of hematopoietic cell proliferation and differentiation. Leukemia is classified both clinically and pathologically as acute or chronic (based on differentiation state and clinical evidence) and myeloid or lymphoid (according to cell type). Extensive deregulation of miRNA has been observed in leukemia, and many studies support its role in aberrant signaling pathways identified in chronic lymphocytic leukemia (CLL), chronic myeloid leukemia (CML), acute lymphoblastic leukemia (ALL), and acute myeloid leukemia (AML).

Chronic lymphocytic leukemia is the most common leukemia in the Western world and is fairly heterogeneous. It can be characterized by IgVH gene mutations, CD38 and ZAP-70 expression, presence of chromosomal abnormalities and p53 dysfunction, causing gradual accumulation of functionally immature B-cells, arrested at G0 or G1 phase (Dohner et al., 2000;Pettitt et al., 2001;Rosenwald et al., 2001;Wiestner et al., 2003;Damle et al., 2007). miRNA variations impact on malignant CLL cells triggering evasion of apoptosis, proliferation, and stimulation of angiogenesis and invasion.Bullrich et al. (2001) andCalin et al. (2002) first reported the involvement of miRNAs in human cancer, identifying a precise region on chromosome 13q14 that contains two miRNA genes, miR-15a and miR-16-1, deleted or down-regulated in about 69% of CLL patients. Via a feedback circuitry, these two miRNAs directly down-regulate tumor suppressor protein TP53, miR-34a, miR-34b, and miR-34c, and increase protein levels of ZAP70 (Fabbri et al., 2011). In CLL patients with 13q deletions, this mechanism is altered with a consequent reduction of CDKN1A, BBC3, and BCL2 expression (Cimmino et al., 2005;Fabbri et al., 2011). Moreover, miRNA expression profiles characterizing CLL phenotype have demonstrated that down-regulation of miR-223, miR-29c, miR-29b, and miR-181 families is strongly associated with disease progression in CLL cases harboring 17p deletion (Visone et al., 2009), while miR-21, miR- 92, miR-101, miR-150, miR-155, miR-146a, and miR-17-92 families are all highly expressed in B-CLL (Calin et al., 2004, 2005;Fulci et al., 2007). In particular, miR-155 and miR-21 are significantly higher in NK cell than in B-cell lymphomas/leukemias, and down-regulate PTEN, PDCD4, or SHIP1 with up-regulation of phosphorylated AKT (ser473;Yamanaka et al., 2009). NFκB, AP1, and MYB transcription factors themselves regulate miR-155 (Vargova et al., 2011).

A further indication of the importance of miRNAs in CLL pathogenesis is given by miR-29 and miR-181 in regulating T-cell leukemia/lymphoma 1 (TCL1) oncogene, overexpressed in 25–35% of CLL patients (Pekarsky et al., 2006). They are down-regulated in cases with 11q/17p deletion and in aggressive CLLs correlating with poor prognosis. In particular, miR-29 family is known to target CDC42, which reduces p53 levels and PI3K activity (Park et al., 2009). miR-181 inhibits BCL-2, MCL-1, and XIAP protein by direct binding to 3′UTR (Zhu et al., 2012). Finally, low expression of miR-34a has been associated with both 17p deletion and chemotherapy resistance in CLLs (Dijkstra et al., 2009;Zenz et al., 2009).

Chronic myeloid leukemia is a disorder marked by an increase in myeloid, erythroid cells, and platelets in peripheral blood with severe myeloid hyperplasia in bone marrow and a translocation on chromosome 9 and 22 (the so-called Philadelphia chromosome) in <95% of CML patients (Croce, 2008). miRNA expression profiles in mononuclear and CD34+ cells from CML patients revealed that miR-10a, miR-150, and miR-151 are downmodulated and miR-96 is up-regulated compared with healthy controls (Agirre et al., 2008). Moreover, miR-17-92 are overexpressed for transactivation induced by both breakpoint cluster region-c-abl oncogene (BCR/ABL) and c-Myc in primary CML CD34+ cells in chronic phase compared with normal CD34+ cells (Venturini et al., 2007), and are regulated by members of the E2 transcription factor family in a negative feedback loop (O’Donnell et al., 2005). (Bueno et al. (2008) found that miR-203 functions as a tumor suppressor and is silenced by hypermethylation in hematopoietic malignancies expressing either ABL1 or BCR/ABL1.Chaubey et al. (2009) detected that miR-219-2 and miR-199b can be hemizygously lost in a significant proportion of CML cases with der (9q) deletion. As tumor suppressor, miR-181a targets RalA associated with cell proliferation, G2-phase arrest, and apoptosis in CML (Fei et al., 2012).Eiring et al. (2010) demonstrated a RISC-independent decoy activity for miR-328, which is down-regulated in CML blast crisis (CML-BC). Loss of miR-328 occurs in CML-BC in a BCR/ABL dose- and kinase-dependent manner through the MAPK-hnRNP E2 pathway. Restoration of miR-328 expression rescues differentiation and impairs survival of leukemic blasts by simultaneously interacting with the translational regulator poly(rC)-binding protein hnRNP E2 and with mRNA encoding survival factor PIM1, respectively. Interaction with hnRNP E2 is independent of the miRNA seed sequence and leads to release of CEBPA mRNA by hnRNP E2-mediated translational inhibition.

Acute lymphoblastic leukemia is one of the most common malignancies observed in the pediatric age group. It is characterized by clonal proliferation of early B- and T-lymphocyte progenitors and results in the accumulation of leukemic lymphoblast in bone marrow and various extra-medullary sites (Crazzolara and Bendall, 2009).Danen-van Oorschot et al. (2012) showed that 14 miRNA genes are up-regulated (miR-128a, miR-142-3p, miR-142-5p, miR-150, miR-181a, miR-181b, miR-181c, miR-193a, miR-196b, miR-30e-5p, miR-34b, miR-365, miR-582, miR-708) and five are down-regulated (miR-100, miR-125b, miR-151-5p, miR-99a, let-7e) in ALL cells compared with normal CD34+ cells. Specific miRNA expression profiles have been defined in major subtypes of ALL (T-cell, MLL-rearranged, TEL–AML1-positive, E2A-PBX1-positive, and hyper-diploid acute lymphoblastic leukemia) and identified as highly predictive of clinical outcome (Schotte et al., 2011). Recently,Mi et al. (2007) identified 27 miRNAs that were differentially expressed in ALL compared with AML; among these, miR-128a and miR-128b were significantly overexpressed, whereas let-7b and miR-223 were strongly down-regulated. miR-128b (higher in ALL vs AML) was also overexpressed in ALL vs normal CD19+ cells. Overexpression of miR-128 in ALL was at least partly associated with promoter hypomethylation and not with an amplification of its genomic locus.

Acute myeloid leukemia is characterized by an accumulation of granulocytic or monocyte precursors in bone marrow and peripheral blood. miRNA patterns have been correlated with cytogenetic and molecular subtypes of AML (Jongen-Lavrencic et al., 2008;Li et al., 2008;Saumet et al., 2009). miRNA expression has also been investigated in some AMLs associated with rare translocations. Interestingly, an elevated expression of miR-125b-1 was observed in AMLs carrying the t(2;11)(p21;q23) translocation (Bousquet et al., 2008). Similarly, miR-125b overexpression causes highly invasive myeloid leukemia, such as BCR/ABL-positive leukemia, and has been associated with drug resistance in TEL–AML1-positive pediatric ALL (Gefen et al., 2010;Schotte et al., 2011). Its leukemogenesis pathway may include down-regulation of IRF4, a transcription factor that inhibits proto-oncogene BCL-6 (B-cell CLL/lymphoma 6) in lymphoma (Saito et al., 2007;Bousquet et al., 2010). Studies have identified other miRNAs whose expression is altered in acute promyelocytic leukemia (APL). Particularly, miR-342 is downmodulated by the binding of PML/RAR-alpha to its promoter in leukemic compared to normal promyelocytes (Careccia et al., 2009) and is up-regulated during APL differentiation upon ATRA treatment (De Marchis et al., 2009).

A recent study provided evidence that some miRNAs are involved in control of DNA methylation machinery, and their deregulation may be partly responsible for aberrant DNA hypermethylation observed in AMLs. Particularly, miR-29b overexpression in AML cells results in a marked reduction in expression of DNA methyltransferases DNMT1, DNMT3A, and 3B leading to a decrease in global DNA methylation and re-expression of genes silenced through hypermethylation. In addition, miR-29b indirectly down-regulates DNMT1 by targeting Sp1 (Garzon et al., 2009). Moreover, DNA methylation analyses of the CpG island of C/EBPα identified a densely methylated upstream promoter region in 51% of AML patients and the silencing of miR-124a by epigenetic mechanisms. This miRNA targets the C/EBPA 3′UTR (Hackanson et al., 2008). Another study showed that miR-29b is involved in a protein–miRNA network including SP1, NFκB, and HDAC, whose deregulation results in KIT overexpression in AML and is associated with adverse clinical outcome (Liu et al., 2010).Fazi et al. (2005) also showed that AML1/ETO oncoprotein triggers heterochromatic silencing of miR-223 transcription by recruiting chromatin-remodeling enzymes (HDAC, DNMT and MECP2) at an AML1-binding site on the pre-miR-223 gene, thus contributing to the differentiation block of AML1/ETO+ myeloid precursors. In myeloid progenitor cells and AML patients with t(8;21),Zaidi et al. (2009) reported that Runx1 and AML1-ETO occupy the miR-24-23-27 locus and reciprocally control miR-24 transcription, enhance growth factor-independent proliferation and block granulocytic differentiation of myeloid cells. Down-regulation of miR-34b caused by promoter methylation has also been explored in AMLs as a possible determinant of an increase in its target cyclic AMP-response element binding (CREB;Pigazzi et al., 2009). In addition, miR-212 has been reported as an independent prognostic factor associated with prolonged overall survival and relapse-free survival (Sun et al., 2012a). Finally, particular emphasis should be focused on the development of next-generation deep-sequencing data for novel miRNAs and both somatic and germline genetic variants of leukemia subtype-specific miRNA gene identification (Calin et al., 2005;Starczynowski et al., 2011). This technique has also applied to accurately measure mature miRNA expression and define their functional role in miRNA stability and processing (Ramsingh et al., 2010). Recent findings showed several novel miRNAs located in leukemia-associated genomic alterations. For example, miR-145 and miR-146a are commonly found in deleted region in del (5q) myeloid malignancies and are down-regulated in cell lines with the chromosome 5q deletion or diploid at this locus compared with CD34 cells. Notably, miR-481, located within a deleted region on chromosome 7q, is able to target meningioma 1 (Mn1). Its higher expression is a predictive factor of poor outcome in patients with AML (Starczynowski et al., 2011). Similarly, miR-223* completes miR-223 function, activating apoptosis and/or inhibiting self-renewal or proliferation of progenitor cells. High expression levels have been associated with a better overall survival rate in AML patients with normal cytogenetics (Kuchenbauer et al., 2011).

## CONCLUSION

Control of gene expression by miRNAs is a finely regulated and complex process able to determine cellular phenotype and fate. Distinctive miRNA expression profiles emerging from the current literature indicate that these patterns might prove useful for the classification of aberrant phenotypes. miRNA alterations seem to actively and profoundly contribute to malignant transformation and progression of cancers, including leukemia. Based on distinctive miRNA signatures in different leukemia networks, miRNAs are proposed as potential biomarkers with considerable impact in diagnosis and prognosis, as well as in detecting cancer at its earliest stages, characterizing specific cancers or defining “patient clusters” evasive or responsive to treatment. In addition, selective modulation of target genes involved in leukemogenesis by miRNA has supported the development of miRNA-based therapeutic strategies. Depending on miRNA function and its status in cancer tissues, new therapeutic approaches have been generated to restore a loss of miRNA function or to inhibit it. In both cases, the reprogramming of miRNA leads to a re-establishment of non-pathological pathways. To date, some miRNA antagonist/mimic-based oncology therapies using modified oligonucleotides and effective delivery systems, such as adeno-associated virus (AAV), cationic liposomes or polymer-based nanoparticles, have been validated in clinically relevant animal models and are currently in pre-clinical development. The systemic delivery of miR-155 antisense encapsulated in polymer nanoparticles (Babar et al., 2012) and miR-34 mimics in ionizable liposome (Bader, 2012) to pre-B-cell tumors *in vivo* seems very promising. The latter is the first miRNA mimic due to enter phase I clinical trials in early 2013 for leukemias and lymphomas (Mirna Therapeutics Inc, 2011). Current advances confirm the potential clinical miRNA-based applications in effective, tolerated and “custom” hematological cancer treatment.

## Conflict of Interest Statement

The authors declare that the research was conducted in the absence of any commercial or financial relationships that could be construed as a potential conflict of interest.
